# Percutaneous treatment of ranulas: ultrasound-guided drainage with salivary gland chemical ablation

**DOI:** 10.1007/s00247-019-04356-x

**Published:** 2019-02-27

**Authors:** Zachary J. Brannan, Lacey J. Lubeley, Sean A. Sutphen, James W. Murakami

**Affiliations:** 1 Department of Radiology, Nationwide Children’s Hospital, 700 Children’s Drive, Columbus, OH 43205 USA; 2 Illinois Bone and Joint Institute, Chicago, IL USA

**Keywords:** Ablation, Children, Ethanol, Ranula, Salivary gland, Sclerotherapy

## Abstract

**Background:**

Ranulas are salivary pseudocysts in the floor of the mouth adjacent to damaged salivary glands. Current surgical management is drainage of the ranula with removal of the offending gland. An analogous percutaneous procedure could potentially offer similar treatment efficacy in a more minimally invasive way.

**Objective:**

To evaluate the outcomes of a cohort of patients with ranulas treated with percutaneous ranula aspiration and chemical ablation of the source salivary gland to see whether this technique could be proposed as a minimally invasive treatment alternative.

**Materials and methods:**

This retrospective single-center study evaluated 24 patients treated percutaneously for ranulas between January 2004 and December 2014. All patients were treated with percutaneous ranula aspiration and chemical ablation of the offending salivary gland. Treatment success and any complications were recorded.

**Results:**

Complete ranula eradication was successfully accomplished in 87.5% of the patients with no complications.

**Conclusion:**

Initial results suggest that our technique of percutaneous aspiration of ranulas and chemical ablation of the source salivary gland is safe and effective.

## Introduction

A ranula is a mucus-filled salivary pseudocyst in the floor of the mouth [1]. A ranula arises from a salivary gland or duct after trauma or blockage [2]. Ranulas most commonly originate from the sublingual gland, but they can also arise from the submandibular gland [3]. A simple or intraoral ranula is located within the floor of the mouth while a plunging or cervical ranula extends inferior to the mylohyoid muscle, often herniating through a defect in the muscle [4, 5].

Ranulas are differentiated from other floor-of-mouth fluid collections based upon clinical presentation and imaging. Symptoms include fluctuating degrees of swelling and pain, intermittent spontaneous leakage into the mouth, and difficulty swallowing, speaking, breathing and chewing [4].

Historically, ranulas were surgically marsupialized or excised, but both practices resulted in high recurrence rates [2, 3, 6, 7]. Current recommended surgical management is removal of the offending salivary gland combined with excision or drainage of the ranula [8]. These procedures, while usually effective, can result in complications [2, 3, 8–11]. Surgical techniques continue to evolve to minimize these complications [12–14].

Alternative practices such as sclerotherapy have been introduced [15–19]. Similar to early surgical techniques, sclerotherapy of the cyst without treatment of the offending gland can have high recurrence rates [18]. In a recent report, only a 45% complete cure rate was achieved when the ranula sac alone was injected with ethanol [19]. We hypothesized that ablating the offending salivary gland in addition to draining the ranula would improve the efficacy of percutaneous treatment. Percutaneous ablation of salivary tissue has been validated in animal models as a means of decreasing gland volume and presumably saliva production [20, 21]. Therefore, to address the clinical problem of a ranula with a minimally invasive procedure that treats both the ranula and the offending salivary gland, we combined image-guided percutaneous ranula aspiration with chemical salivary gland ablation.

We report our experience treating ranulas in 24 patients using our two-component technique.

## Materials and methods

This retrospective study was reviewed and approved by our Institutional Review Board and informed consent was waived. We retrospectively reviewed our institutional experience with this procedure between 2004 and 2014 to assess its outcomes.

We treated 67 pediatric and adult patients with ranulas between January 2004 and December 2014. Of those, 24 met our inclusion criteria and were included in this analysis. Our inclusion criteria were that the patients needed to be 21 years old or younger, had to have imaging as well as clinical diagnostic confirmation of diagnosis before treatment and had to have returned for follow-up appointments with imaging confirmation of outcome. In order to remove as many confounding variables as possible, patients older than 21 years, those who previously failed surgical treatment(s) and those lost to follow-up were excluded from this analysis. Patient demographics, lesion size, treatments received and outcomes are outlined in Table 1. All patients clinically presented with visible or palpable swelling in their mouths and/or necks noticed by themselves, their families or their physicians.

**Table 1:** Patient demographics and outcomes

Patient	Age (years)	Gender	Side	Type	Originating gland	Maximum diameter (cm)	EtOH (mL)	STS (mL)	# of treatments	Total follow-up (months)	Recurrence at last follow-up
1	12	M	R	S	SLG	1.7	1.5	1.5	1	2	No
2	19	M	R	P	SLG	4.1	3	-	1	13	No
3	7	F	R	P	SLG	1.9	3, 3	-	2	4	No
4	5	F	L	P	SMG	2.1	5	-	1	1	No
5	12	F	L	S	SLG	1.3	3	-	1	2	No
6	6	M	R	P	SLG	4.1	5, 3	-	2	24	No
7	11	F	R	P	SLG	3.5	2, 4.8, 2	-	3	22	No
8	13	M	R	S	SLG	3	1.5	-	1	3	No
9	2	F	R	P	SLG	2.6	1.8, 3	-	2	4	No
10	21	F	R	S	SLG	4.7	2	1.5	1	28	Yes
11	5	F	R	S	Unclear*	2.4	3.5, 3	0, 4	2	58	Yes
12	4	M	R	S	SLG	2.3	3	-	1	1	No
13	9	F	L	P	SLG	2.6	3	-	1	37	No
14	15	M	R	S	SLG	1.9	2	-	1	1	No
15	9	F	R	S	SLG	0.8	2	-	1	1	No
16	7	F	R	S	SLG	0.9	3	-	1	1	No
17	18	M	R	S	SLG	1.9	3, 1.5	0, 1.5	2	13	No
18	15	F	L	P	SLG	3	3, 4	0, 2	2	11	No
19	12	F	R	S	SLG	2.1	3	-	1	1	No
20	1	M	L	S	SLG	1.8	2	-	1	38	No
21	15	F	L	P	SLG	5.8	1.2	2	1	3	No
22	7	F	R	S	Unclear*	2	3.5	4	1	7	No
23	5	F	L	S	SLG	1.6	4	-	1	54	No
24	1	M	R	S	SLG	1.7	2.5	0.5	1	4	Yes

*SMG and SLG treated

*EtOH* ethanol, *F* female, *L* left, *M* male, *P* plunging, *R* right, *S* simple, *SLG* sublingual gland, *SMG* submandibular gland, *STS* sodium tetradecyl sulfate

The final cohort consisted of 9 females and 15 males ranging in age from 1 year to 21 years with an average age of 9.6 years. Fifteen were simple ranulas and nine were plunging ranulas. Seventeen patients had right-side ranulas.

**Fig. 1:**
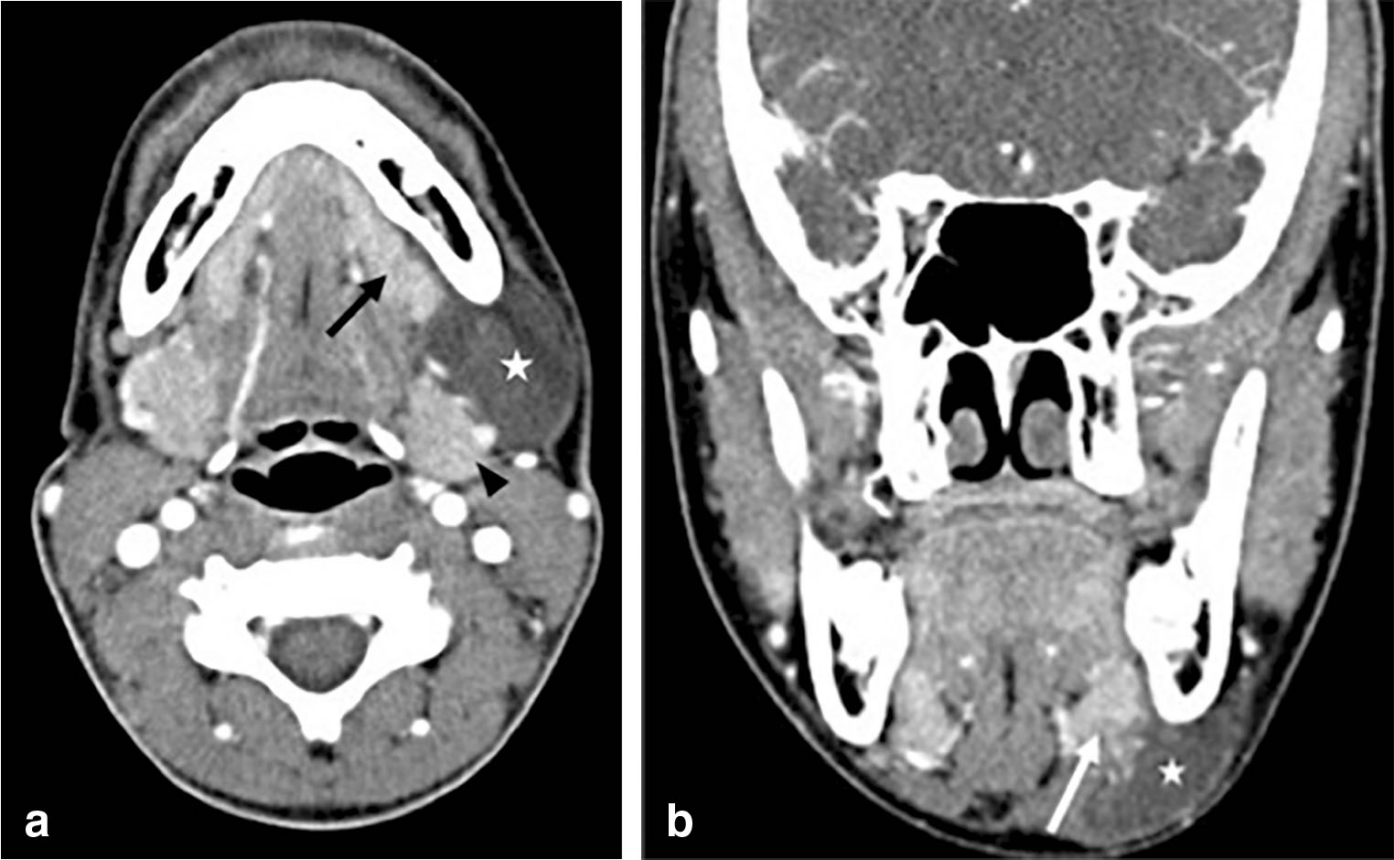
Contrast-enhanced computed tomography images of a typical simple ranula in a 12-year-old girl: Axial (**a**) and coronal (**b**) images show the ranula (*star*) between the likely source of the salivary leak, the sublingual gland (*arrow*), and the body of the left hemi-mandible. The coronal image (**b**) shows the left sublingual gland (*arrow*) larger than the right and with heterogenous lower attenuation inflamed edge abutting the ranula (*star*). The axial image (**a**) also shows how the ranula can abut both the sublingual gland (*arrow*) and the submandibular gland (*arrowhead*)

Patients were diagnosed before referral to Interventional Radiology based upon the clinical presentation of characteristic intraoral swelling or by cross-sectional imaging such as ultrasound (US), computed tomography (CT) or magnetic resonance imaging (MRI) (Fig. 1). Before their referral to Interventional Radiology, 3 patients had no imaging, 17 patients had diagnostic US, and 9 had CT and/or MRI. All ranulas were documented with US at the time of treatment.

All procedures were performed in the Interventional Radiology suite by two attending interventional radiologists (William E. Shiels, with approximately 20 years of clinical experience, and J.W.M., with approximately 15 years of clinical experience). All patients were treated under general anesthesia or conscious sedation on an outpatient basis.

**Fig. 2:**
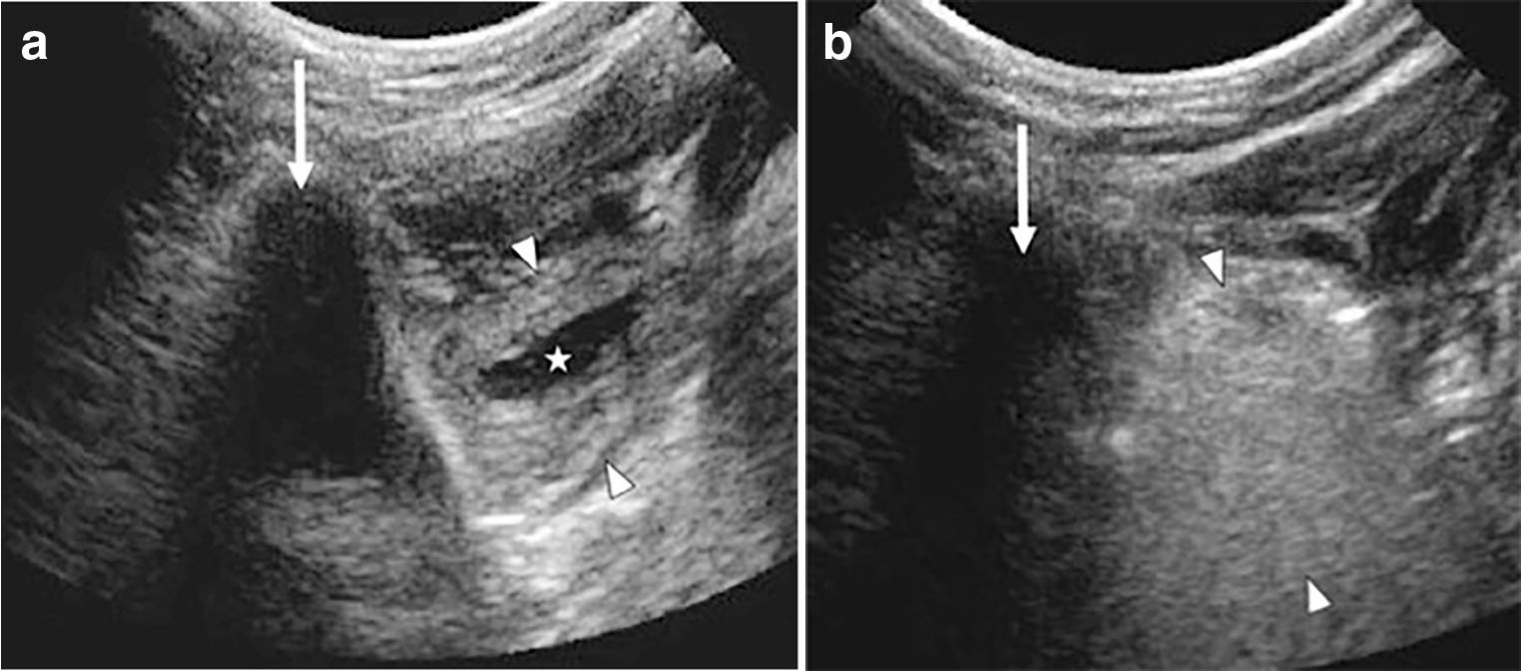
Coronal ultrasound images taken through the floor of the mouth during ranula treatment in a 10-year-old boy. Image orientation during treatment is from the skin surface under the chin scanning up into the mouth. To help with image orientation, *arrows* identify the shadowing mandibular body adjacent to the sublingual gland in both images. The sublingual gland (*arrowheads*) before treatment with a cleft (*star*) indicates the likely source of the salivary leak. This cleft is rarely seen but is a good indicator that this gland is the offending gland. When injecting in this area, fluid can sometimes be seen to track from the fissure to the ranula. The appearance of the sublingual gland (**b**) during alcohol injection (*arrowheads*). As with alcohol injection in other tissues, the gland becomes vibrantly hyperechoic during and immediately after alcohol injection

After examining the patient and the images, the diagnosis of ranula was made and the likely gland of origin identified. In 21 patients, the sublingual gland was the obvious gland of origin. In the remaining three patients, the submandibular gland was the gland of origin or it was simply unclear as the ranula resided directly between the sublingual gland and submandibular gland, abutting both glands (Fig. 1). The gland of origin was sometimes implicated by associated structural damage or inflammatory changes seen on pre-procedural imaging (Figs. 1 and 2). We used US, CT or MRI findings, such as heterogeneous echotexture, swelling, gland margin irregularity or internal fissuring, or altered enhancement with CT and MRI to help identify the potential gland of origin.

Once prepped, the ranula sac was punctured and aspirated under US guidance with a 14-gauge angiocatheter. The characteristic thick yellow fluid was discarded and was not sent for any diagnostic tests. In our experience, smaller needles are of insufficient caliber to aspirate the thick mucous of most ranulas. Under constant US guidance, using a percutaneous approach from under the chin and into the floor of the mouth, the suspected salivary gland of origin was punctured with a 22-gauge or smaller needle and sclerosant was injected into the gland. Usually, this meant that the sublingual gland was injected (Fig. 2). If the source of the leak was felt to be the submandibular gland or if it simply was unclear which gland was the source, the sublingual gland and the surface of the submandibular gland abutting the ranula were both injected. The submandibular gland can be relatively large and during the time of this study, rather than injecting the entire submandibular gland, we infiltrated the portion of the submandibular gland that appeared by US to be inflamed or fissured or was in immediate contiguity with the ranula sac.

Early in our experience, 98% dehydrated alcohol (EtOH) (American Regent Inc., Shirley, NY, USA) alone was injected (1.2–5 mL). The operator estimated the quantity of any agent injected at the time of the procedure and included it in the procedure report and nursing record. If the ranula recurred, the gland was treated in a second procedure with a combination of 3% sodium tetradecyl sulfate (STS) (Mylan Institutional LLC, Rockford, IL, USA) (0.5–5 mL) followed by EtOH (1–5 mL). It is our impression that injection of STS works synergistically with EtOH to increase cell toxicity. This impression, together with our subjective observation of greater clinically visible inflammation achieved when we used both STS and EtOH for recurrent disease, led us to use both during all treatments toward the end of this study and is our current standard of practice. If both STS and EtOH were injected, the following sequence was used. First, 3% STS was injected throughout the target gland to a volume needed to infiltrate the gland, which swells and becomes more heterogeneously hypoechoic and fissured. Following a delay of a few minutes to get the next syringe ready, the EtOH is injected to a volume needed to infiltrate the target turning it diffusely hyperechoic (Fig. 2). Usually when injecting a sublingual gland, it requires 2–3 punctures to infiltrate the gland given its long thin shape. When injecting a submandibular gland, 1–3 punctures may be needed. Many times a single capsular puncture can be made into the submandibular gland and, using a fan-shaped injection technique, the needle can be moved throughout much of the gland without removing it from the capsule of the gland.

Once recovered from anesthesia or sedation, all patients were discharged to home. There were no incisions so no postoperative wound care was necessary and antibiotics were not routinely used. No activity restrictions or additional therapy or diet were needed post procedure. Any complications immediately after the procedure or during follow-up were recorded.

Outcomes were assessed during clinical follow-up visits. US imaging was used at follow-up appointments to evaluate treatment efficacy. No other follow-up imaging was obtained though some patients had additional head and neck imaging for other clinical indications later. If the ranula was completely resolved and no residual clinical or imaging abnormality remained, the treatment was considered a success. If any portion of the ranula sac was visible by US at follow-up, the procedure was considered a failure. If the procedure failed to completely eradicate the ranula, a repeat procedure was performed in most patients. After one or more failed treatments, patients could elect to proceed with open operative ranula surgery. The only statistical measure recorded was the percent of patients who achieved complete cure with our treatment relative to the 24 patients we treated.

## Results

Outcomes were assessed during clinical follow-up visits ranging from 3 weeks to 58 months after treatment with an average of 13.9 months. Twenty-one of the 24 patients (87.5%) were successfully treated with ranula sac aspiration and salivary gland ablation (Table 1). Fifteen patients (62.5%) were successfully treated with one sclerotherapy procedure. Five patients (20.8%) required two treatments and one patient (4.1%) required three treatments. In our small sample, there were no identifiable differences between patients needing multiple treatments and the others with respect to their clinical features, their lesions or amounts of sclerosants injected. The remaining three patients (12.5%) proceeded to open surgical salivary gland removal after the first or second failed percutaneous treatments (Table 1). All recurrences occurred within several months of treatment except for two that occurred 28 months and 58 months after their last treatment. Both of these patients were referred for operative management as we were concerned that there might be something anatomically or pathologically confounding in these glands causing recurrence that would be better served with operative resection of the entire gland. Of the 32 procedures performed, 24 were performed with EtOH alone and 8 were performed with the combination of STS and EtOH. In our patient cohort, the average ranula sac size was 2.5 cm and was not associated with the type of treatment chosen or the outcome (Table 1).

The volume of alcohol injected during each treatment reflects what was injected into the sublingual gland if that was all that was treated or both the sublingual gland and the submandibular gland when both were injected during a single treatment as was the case for recurrent ranulas. The amount of alcohol used during any single treatment ranged between 1.5 and 5 mLs over the 32 treatments for an average of 2.8 mL. These are estimates by the operator and reflect individual doctor preference for volumes as well as different-sized patients and glands. No attempt was made at the time of the procedures to critically record why certain volumes were used in each patient. The goal was to visualize treatment-related echogenic changes during US-guided injection throughout the target gland while minimizing any non-target organ ablation. Alcohol was injected until the entire targeted gland or area of a gland was rendered completely echogenic. When STS was injected before the alcohol, the volumes were similar.

Expected post-sclerotherapy swelling resolved in less than 2 weeks in all patients and never resulted in hospitalization or difficulty breathing, eating or speaking. In this cohort, there were no complications such as skin or nerve injury.

## Discussion

Ranulas are salivary pseudocysts resulting from saliva extravasation out of a damaged salivary gland or duct [1]. As pseudocysts, they are lined by connective tissue rather than epithelium [1]. The exact cause of the extravasation is not always clear, but the general consensus is that a discrete unit or lobule within a salivary gland leaks saliva into the surrounding tissues forming a pseudocyst [9, 22, 23]. Plunging ranulas arise when a simple ranula herniates or erodes through a dehiscence in the mylohyoid muscle [4, 24, 25].

While simple intraoral ranulas are easily diagnosed by history and physical exam, deeper ones benefit from cross-sectional imaging such as US, CT or MRI to differentiate them from other diagnostic considerations and to potentially define the gland of origin. We primarily use US, which is an effective, inexpensive and nearly universally available tool to diagnose patients without exposure to radiation or injected contrast. For complex or diagnostically confusing cases, particularly plunging ranulas, CT and MRI help to confirm the diagnosis with better anatomical detail [26]. The extremely viscous yellow fluid of a ranula, if sent for laboratory evaluation, can be positive for amylase and mucin [27]. We find that clinical presentation, imaging and the very characteristic gross appearance of aspirated fluid is sufficient for diagnosis and do not routinely send the aspirated fluid for laboratory evaluation.

Surgical experience has shown that ranula cyst excision or marsupialization without removing the offending salivary gland has variable but always high recurrence rates of 37–100% [2, 5–8]. To surgically cure a ranula, it is necessary to remove the responsible salivary gland [3, 8, 22]. Recurrence rates using this surgical approach for intraoral ranulas are low, ranging between 0% and 3.6%, and likely result from incomplete removal of the offending damaged glandular tissue [2, 3, 6, 7]. Higher rates of recurrence can be expected with plunging ranulas [22].

Both intraoral and cervical approaches can be used for surgical excision. The intraoral approach is more technically demanding while the cervical approach is more invasive with higher potential for complications [28]. Morbidity associated with surgery includes injury to the facial nerve, lingual nerve, marginal mandibular nerve, Wharton’s duct, submandibular gland and vascular structures, as well as postoperative bleeding and infection [2, 8, 9, 11]. Surgical studies have reported lingual nerve damage in up to 11% of patients, and damage to Wharton’s duct requiring additional surgery in 1–2% [5, 8, 11, 15, 22]. To decrease surgical invasiveness and minimize complications, new surgical procedures have been proposed including altering incision locations or performing partial gland-sparing surgeries to minimize injury to adjacent structures [12–14, 29, 30].

With the goal of decreasing invasiveness even further, sclerotherapy has been reported for the treatment of ranulas where the cysts are aspirated and injected with caustic or inhibitory agents such as OK-432 and EtOH [15–19, 31–33]. There are a variety of different sclerosing agents available but the most reported agent used for ranula treatment is OK–432 [15–18]. OK-432 sclerotherapy of the ranula cyst requires multiple treatments, each associated with a significant amount of inflammation necessary for a robust fibrotic response [17, 18]. As the ranula sac is a pseudocyst and its wall or margin is not the source of the fluid, sclerotherapy would not be expected to have much effect on the actual problem unless the agent extended from the sac back into the source gland. Complete ranula eradication with OK-432 has been reported at 33% with an overall recurrence rate after each injection of 47% [18]. Likewise, complete ranula eradication with ethanol has been reported at 45% [19].

Ideally, a less invasive treatment such as sclerotherapy needs to yield a similar outcome, complete cure, to the more invasive surgical solutions with a decrease in perioperative discomfort, recovery time and operative risk. We sought to improve upon published sclerotherapy results by incorporating the surgical treatment paradigm of treating the offending salivary gland rather than solely addressing the ranula sac. In our treatment protocol, we chose to chemically ablate the offending salivary gland and aspirate the ranula sac. Once the source of the saliva is treated there should be no need to treat the wall of the pseudocyst. Avoiding injection of a sclerosant into the cyst would obviate any risks with that action. We chose to aspirate the cysts as that is usually trivial to do during the procedure and gives the patients immediate relief from any symptoms related to the ranula sac such as swelling or altered tongue position.

In this study, we successfully treated and completely cured 87.5% of the patients, an increase from prior complete cure rates of 33% and 45% discussed above for other percutaneous techniques [18, 19]. Six of the 21 successfully treated patients needed more than one session. Fortunately, the nature of the intervention is less invasive than surgical options and this was well tolerated. Comparing our outcomes with surgical alternatives is not possible as there are simply too many variants of surgical management, but we believe a nearly 90% success rate is comparable to those reported in surgical series [2, 3, 5, 6, 10, 11]. Comparing the postoperative recovery from our procedure with that of surgical series is not possible as the surgical techniques vary and this aspect of the surgical treatment is commonly not reported. What can be said is that open surgery is usually more invasive than our technique and often requires more involved wound care and drain placement [5, 12, 14]. All our cases were performed on an outpatient basis with no need for any postoperative wound care, no need for any drains and with only mild perioperative pain and swelling allowing a rapid return to normal activity usually within a day or two. Lastly, as with intraoral surgical techniques, there is no external scarring [5].

In this study, there were no complications related to any of the 32 procedures performed. The safety and efficacy of ethanol for sclerotherapy of cystic and solid structures have been well established [34–36]. Based upon experience treating cystic and solid lesions with alcohol, we certainly recognize the risks of non-target tissue injury with temporary muscle weakness [36]. Our own 15-year experience with injecting alcohol into the sublingual gland and submandibular gland for hypersalivation has taught us that it can result in temporary injury to lingual and marginal mandibular nerves with resultant paresis of subtended muscles. Therefore, though we saw no nerve injuries in this reported cohort, one should anticipate this complication to occur occasionally with the procedure. But this has to be interpreted in light of documented risks of open surgical treatments that include permanent nerve injury [2, 8, 11]. Given the different surgical techniques, each with a slightly different risk profile, and our small sample size with no complications, it is impossible to do any kind of side-by-side comparison of complications.

The present study has limitations not new to Interventional Radiology and those are its retrospective nature and small sample size. A prospective comparison of this to the other treatment options is unlikely to be feasible as there are so many surgical variations possible all with different pros and cons. Our sample size is limited by the inclusion criterion for the study that mandated imaging follow-up for confirmation of complete eradication of the ranula. We felt imaging confirmation was necessary for this report on our early experience with this technique. Follow-up imaging is not often necessary in asymptomatic patients as recurrences are usually clinically obvious. Likewise, clinical follow-up beyond several months is not necessary as most patients recur in that time frame and can be relied upon to seek reevaluation should symptoms recur at a much later date. We had two patients present with clinical recurrences 28 and 58 months after their last treatment. Extremely long-term follow-up would be needed to completely exclude other recurrences in our cohort. Our clinical practicalities therefore limit this retrospective study so that it lacks the scientific rigor of a prospective study that would include multiyear clinical and imaging follow-up. For now, we believe this study is sufficient to introduce this technique as a safe and effective treatment alternative.

## Conclusion

We have presented a percutaneous, minimally invasive technique for treatment of ranulas that keeps with successful surgical practice by treating not only the ranula sac but also the salivary gland of origin. Our rates of complete success (87%) are close to those from reported surgical series (95–100%) with a decrease in operative complexity and potential risk. We believe it is a viable treatment alternative that is less invasive than most available surgical options.
